# *In vivo* dynamics of active edema and lethal factors during anthrax

**DOI:** 10.1038/srep23346

**Published:** 2016-03-21

**Authors:** Clémence Rougeaux, François Becher, Eric Ezan, Jean-Nicolas Tournier, Pierre L. Goossens

**Affiliations:** 1 Unité Interactions Hôte-Agents pathogènes, Institut de Recherche Biomédicale des Armées, Brétigny-sur-Orge, France; 2 Pathogénie des Toxi-Infections Bactériennes, Institut Pasteur, Paris, France; 3 CEA, iBiTec-S, Service de Pharmacologie et d’Immunoanalyse, 91191 Gif-sur-Yvette, France; 4 CEA, DRF, Programme Transversal Technologies pour la Santé, 91191 Gif-sur-Yvette, France; 5 Ecole du Val-de-Grâce, Paris, France

## Abstract

Lethal and edema toxins are critical virulence factors of *Bacillus anthracis*. However, little is known about their *in vivo* dynamics of production during anthrax. In this study, we unraveled for the first time the *in vivo* kinetics of production of the toxin components EF (edema factor) and LF (lethal factor) during cutaneous infection with a wild-type toxinogenic encapsulated strain in immuno-competent mice. We stratified the asynchronous infection process into defined stages through bioluminescence imaging (BLI), while exploiting sensitive quantitative methods by measuring the enzymatic activity of LF and EF. LF was produced in high amounts, while EF amounts steadily increased during the infectious process. This led to high LF/EF ratios throughout the infection, with variations between 50 to a few thousands. In the bloodstream, the early detection of active LF and EF despite the absence of bacteria suggests that they may exert long distance effects. Infection with a strain deficient in the protective antigen toxin component enabled to address its role in the diffusion of LF and EF within the host. Our data provide a picture of the *in vivo* complexity of the infectious process.

*Bacillus anthracis*, the etiologic agent of anthrax, is a Gram-positive spore-forming bacterium. It is considered one of the most potent and dangerous bioterrorist agents, as shown by the anthrax letter attacks in the fall of 2001.

In rodent models of subcutaneous infection, once in the host, spores rapidly germinate and establish infections at the initial site of inoculation^1^,^2^. Infection progresses to the local draining lymph nodes (dLNs) and the spleen, ultimately the lungs, leading to death^1^,^3^,^4^.

The vegetative form of *B. anthracis* produces a capsule as protection from the host immune defenses, and three, individually non-toxic, proteins that associate to form toxin complexes: lethal toxin (LeTx) which consists of the association of lethal factor (LF) and protective antigen (PA), and edema toxin (EdTx) formed by the association of edema factor (EF) and PA. PA mediates the entry of LF and EF into the cytosol of eukaryotic cells by hijacking two receptors ANTXR1/TEM-8 (Tumor Endothelial Marker-8), and ANTXR2/CMG-2 (Capillary Morphogenesis Protein-2)^5^,^6^. LF is a zinc dependent metalloprotease that cleaves and inactivates most mitogen-activated protein kinase kinases (MAPKKs)^7^,^8^ disrupting major eukaryotic cell functions^9^. EF is a calmodulin and calcium dependant adenylyl cyclase, which catalyses the conversion of ATP to cAMP^10^,^11^, which induces multiple gene expression alterations through PKA and CREB^12^,^13^,^14^,^15^.

It is delicate to get a precise picture of the *in vivo* effects of LeTx and EdTx, translating from the biochemical level up to the cell, the organ and whole host level^16^. At the cellular level, toxins dismantle the innate and the adaptive immune defenses of the host by acting on macrophages, monocytes, dendritic cells, PMNs, T and B cells^17^,^18^. Consistent with this, *in vivo* studies showed that both toxins critically impair myeloid cells in order to evade the scavenging functions of neutrophils and successfully establish infection^19^. These data suggest that *B. anthracis* relies on the toxins to invade its host. At the organ and animal levels, EdTx and LeTx critically target two distinct vital systems; EdTx induces mortality through hepatocytes, while LeTx kills by acting on cardiomyocytes and vascular smooth cells^20^.

Although numerous studies have been published on intoxication models (i.e. survival after toxin injection), little is known about the physiological level of toxins produced by bacteria along the infection. The first data concerned the rabbit and the guinea pigs, in the late stages of infection^21^,^22^. In these studies, Western blot and engineered immunoassays were used to quantify *B. anthracis* factors.

Recently, several approaches have been developed to detect and more sensitively quantify the enzymatic activity of active factors of *B. anthracis*. LF detection by mass spectrometry (MS) detection of its enzymatic activity has been applied to infected rhesus macaques and human^23^,^24^. In parallel, we have developed a competitive enzyme immunoassay to quantify the amount of enzymatically active EF in the plasma and in the ear cutaneous tissue with a limit of detection (LOD) of 1 to 10 pg/ml according to the animal species 25. All these techniques are highly sensitive and specific and now allow us to envision detection and quantification of active LF and EF actually produced during the entire infectious process.

In this study, we characterised the production of active LF and EF *in vivo* in immuno-competent mice by exploiting the discriminative power of bioluminescence imaging (BLI) to synchronise the analysis of the infectious process with a wild type encapsulated toxinogenic *B. anthracis* strain, defining stages of infection from the asymptomatic to the terminal stage. The role of PA in LF and EF diffusion was also tested through infection with a PA-deficient strain. We focused here on the dynamics of the levels of LF and EF produced locally at the site of infection, in the cutaneous tissue of the ear, and at distance in the blood, thus providing, for the first time, novel information about the complete kinetics of toxin production throughout anthrax infection. Moreover, we show that both LF and EF can be detected in the blood before bacterial dissemination, suggesting that anthrax toxins may exert long distance effects even at the first stages of the infection.

## Results

### Characterisation of the thresholds for LF and EF detection

To understand in depth how *B. anthracis* subverts the host defenses through its toxins, we used highly sensitive assays to quantify the amount of active edema and lethal factors at specific time points of infection, either at the initial site of a cutaneous infection or at distance in plasma. The products of their enzymatic activity was quantified as previously reported^23^,^24^,^25^, by MS for LF (23,24 and this study) and by a competitive immunoassay for EF^25^.

In a first step, we characterised the threshold for the enzymatic quantification of LF and EF by using plasma and ear tissue samples derived from mice infected with an encapsulated *B. anthracis* strain that does not produce any LF and EF (EF- and LF-deleted 9602LC strain). This strain is a phenotypic equivalent of the PA-deleted encapsulated 9602P strain, as it does not produce any toxin, due to a lack of EF and LF synthesis. Its LD50 was less than 40 spores by subcutaneous route in OF1 mice, thus similar to the 9602P strain^4^. As the dynamics of infection in mice are similar for the PA-deleted strain and the wild-type strain^4^, this LF- and EF-deleted strain thus represents an ideal control to set up the detection threshold in the infected samples during an actual equivalent infectious process. The basal values obtained in the plasma and the ear cutaneous tissue for the LF and EF assays were determined at an early (1 h) and a late time point (41 h) of infection with this LF- and EF-deleted strain (Table 1, Supplementary data). The highest values obtained for each sample type was considered as the threshold of detection of LF and EF. The thresholds of detection for EF were thus determined at 2.5 pg/ml (n = 16) in the plasma and 0.34 pg/ear (n = 24) in the ear cutaneous tissue; for LF, at 400 pg/ml (n = 21) in the plasma and 16 ng/ear (n = 24) in the ear cutaneous tissue.

### Bioluminescence to synchronize analysis of cutaneous anthrax infection

**Figure 1:**
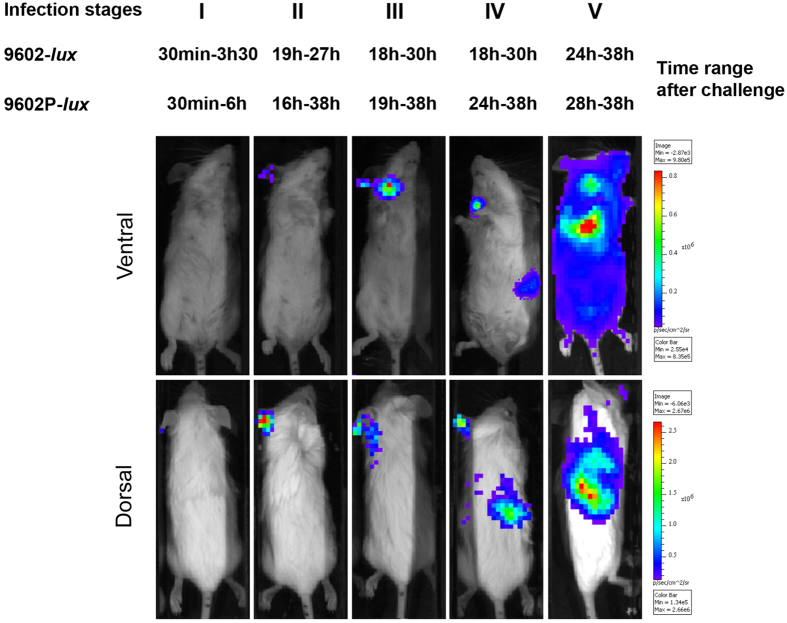
Cutaneous anthrax is stratified along five stages through the time pattern of bioluminescent signalling. BALB/c mice were infected into the ear pinna with spores of the bioluminescent *B. anthracis* strains - 2.73 ± 0.32 log_10_ wild type 9602-*lux* (mean ± SD, n = 24) or 2.46 ± 0.35 log_10_ *pagA*-deleted 9602P-*lux* (mean ± SD, n = 19), as described in the Material and Methods section - and the dynamics of infection followed for each mouse through BLI. Images in this figure depict photographs overlaid with false-colour representations of luminescence intensity, where red is most intense and blue is least intense. Top row: ventral view of a single representative mouse at the indicated time range after infection. Bottom row: dorsal view of the same mouse. Five stages were distinguished (noted I to V) with the time range for all the mice belonging to each stage. The earliest time of infection was associated with absence of BLI (stage I). Stages II, III and IV corresponded to BLI signal displayed in specific organs targeted by *B. anthracis*, i.e. the inoculated ear (stage II), the efferent draining cervical lymph nodes (stage III) and the spleen (stage IV). The final stage of infection associated with lung BLI and septiceamia was noted stage V. The image series are representative of at least 5 independent animal groups for each time range after challenge.

To be in the closest possible physiological setting, immuno-competent mice were infected cutaneously with a fully virulent wild-type *B. anthracis* strain, i.e. encapsulated and producing both toxins. As *B. anthracis* infection does not develop along the same kinetics in each mouse of a same experimental group, despite highly similar experimental conditions, we decided to exploit the discriminative power of BLI to assign each infected mouse to a defined stage of infection according to BLI pattern, i.e. to location and progression of the bioluminescent wild type-*lux* 9602 strain (9602WT-*lux*^4^), or its toxin-deficient derivative 9602P-*lux* strain^26^ in the host (Fig. 1). In this way, the asynchronous character of the infectious process could be controlled and data could be exploited in a homogenous way. Furthermore, the asymptomatic stages of the infection could be approached. Five stages were thus defined:

Stage I corresponded to early infection, when the bacterial load remained below the detection threshold of the bioluminescent signal in the inoculated ear pinna - time range 30 min-3 h 30 and 30 min-6 h for the 9602WT-*lux* (n = 33 mice) and 9602P-*lux* (n = 36) strains respectively.

Stage II was defined by detection of BLI signal exclusively at the initial site of infection, i.e. the ear pinna - time range 19 h- 27 h and 16 h- 38 h for the 9602WT-*lux* (n = 28) and 9602P-*lux* (n = 21) strains respectively.

At stage III, local dissemination to the cervical dLNs was observed, as they displayed BLI - time range 18 h-30 h and 19 h-38 h for the 9602WT-*lux* (n = 31) and 9602P-*lux* (n = 14) strains respectively.

Stage IV corresponded to systemic dissemination, when a BLI signal was detected in the spleen, without signal in the lungs - time range 18 h-30 h and 24 h-38 h for the 9602WT-*lux* (n = 10) and 9602P-*lux* (n = 9) strains respectively.

Stage V coincided with the terminal phase of infection, when mice displayed BLI in the lungs, were septicaemic and displayed the first clinical signs of illness (immediately before ethical euthanasia) - time range 24 h-38 h and 28 h-38 h for the 9602WT-*lux* (n = 13) and 9602P-*lux* (n = 7) strains respectively.

At each stage of infection, quantification of active EF and LF, based on their enzymatic activity, was performed in the inoculated ear cutaneous tissue and the plasma in parallel with the bacterial load.

### Dynamics of LF and EF production at the initial site of cutaneous infection with the 9602WT-lux *B. anthracis* strain

**Figure 2:**
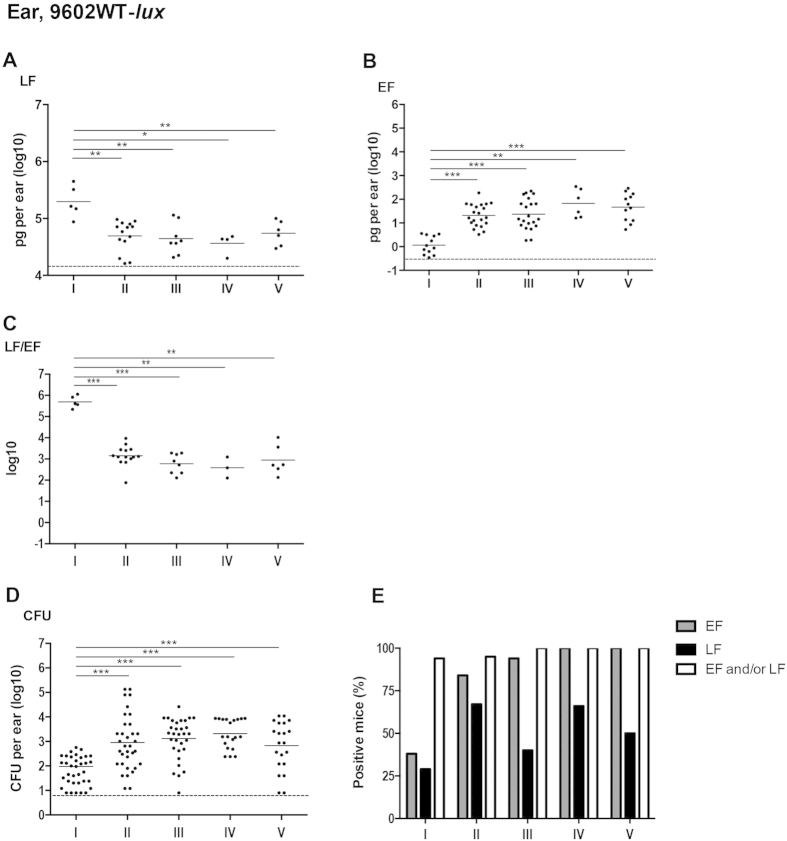
*In vivo* dynamics of LF and EF production at the initial site of infection with the wild type encapsulated toxinogenic 9602-*lux B. anthracis* strain. Active lethal factor (LF, **A**) and edema factor (EF, **B**) and bacterial load (CFU, **D**) were quantified in the inoculated ear cutaneous tissue as described in the section Materiel and Methods, for each stage of infection with the wild type *B. anthracis* 9602-*lux* (9602WT-*lux*) strain as defined in Fig. 1 and in the Results section. The LF/EF ratio (**C**) was calculated from the LF and EF values obtained for each individual mouse. (**E**) Percentages of mice for which the EF or LF value was above the detection threshold (see Table 1, the Materials and Methods and the Results sections); also shown is the combined percentage of mice for which the LF and/or EF values were positive; all mice were positive for *B. anthracis*. Threshold values are represented as dotted lines (0.9 log_10_ for CFU, 16 ng/ear of homogenized ear and 0.34 pg/ear of homogenized ear for LF and EF respectively; see Results section). Results are expressed as pg per total cutaneous ear tissue (pg/ml of homogenized ear); each dot represents an individual mouse and the bar represents the mean for each mouse population at each infection stage. The asterisks denote statistically significant differences (Mann-Whitney test); *p < 0.05; **p < 0.01; ***p < 0.001.

At stage I of infection, 29% of mice (n = 5/17) were positive in the ear pinna, i.e. the site of initial infection, for LF detection and 38% (n = 12/32) for EF detection (Fig. 2E). More importantly, when data were combined, 94% of mice were positive either for EF and/or LF (n = 16/17). At stage II, 67 to 84% of the infected mice were positive for LF or EF respectively (95% when combined). While EF was detected in almost all mice from stage III to V, LF detection was variable as some mice were classified as negative due to the high threshold for LF detection defined in this tissue (Table 1).

At stage I, LF concentration was circa 198 ng/ear (Fig. 2A; Table 2), whereas EF concentration was very low at circa 1.2 pg/ear (mean, n = 12) (Fig. 2B; Table 2). The LF/EF ratio was thus 320,000 (mean, n = 5) at stage I, related to the extremely low level of EF and the high level of LF (Fig. 2C; Table 3). At stage II of infection, the bacterial load in the ear tissue increased (Fig. 2D). Local LF amount in the ear tissue decreased slightly to a plateau of around 45 ng/ear (Fig. 2A; Table 2), while EF concentration increased by 16-fold (Fig. 2B; Table 2), reaching circa 21 pg/ear. This led to a 230-fold decrease in the LF/EF ratio (Fig. 2C; Table 3) to about 1,400 (n = 14). Thereafter, the bacterial burden, and the EF and LF amounts plateaued from stage III (bioluminescent signal in the dLN) to V (terminal septicemia). The LF/EF ratio remained between 400 and 900 (Table 3).

High quantities of LF were thus detected locally at an early time point of the infectious process, whereas EF concentration increased later, its level always remaining lower than that of LF.

### Early detection of LF and EF in the plasma during cutaneous infection with the 9602WT-lux *B. anthracis* strain

**Figure 3:**
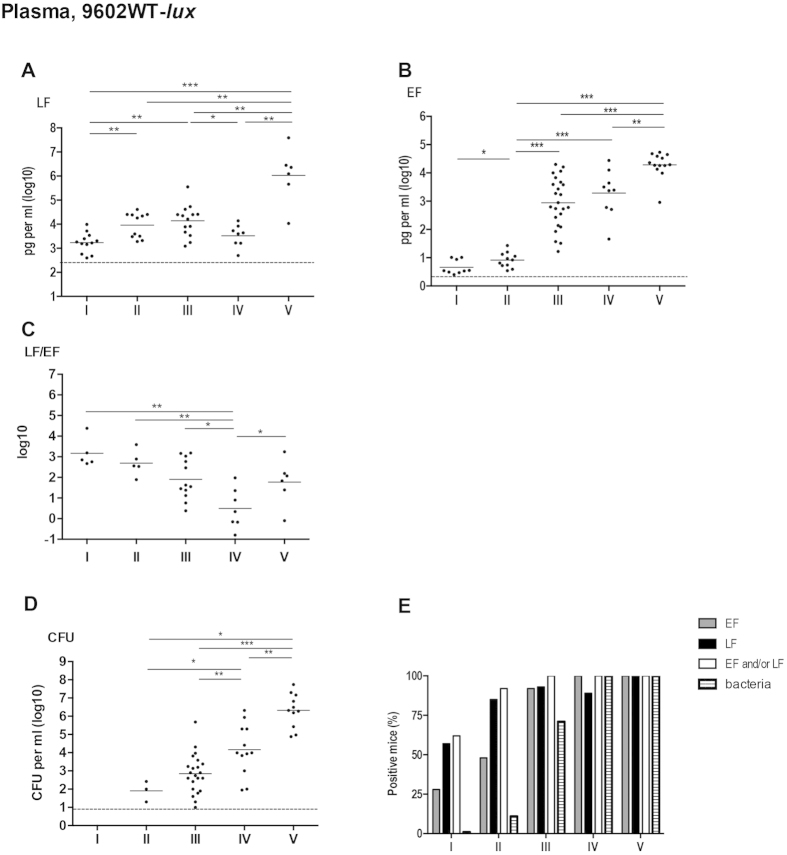
Early detection of plasma circulating LF and EF during cutaneous infection with the wild type 9602WT-*lux* strain. Same experimental conditions as described in Fig. 2. For each infected mouse, blood was collected and LF (**A**), EF (**B**) and bacterial CFU (**D**) were quantified in the plasma. The LF/EF ratio (**C**) was calculated from the LF and EF values obtained for each individual mouse. (**E**) Percentage of mice for which the EF or LF value was above the detection threshold (see Table 1, the Materials and Methods and the Results sections); also shown is the combined percentage of mice for which the LF and/or EF values were positive and the percentage of bacteriaemic mice. Threshold values are represented as dotted lines (0.9 log_10_ for CFU, 0.4 ng per ml and 2.5 pg/ml for LF and EF respectively; see results section). Results are expressed as pg per ml; each dot represents an individual mouse and the bar represents the mean for each mouse population at each infection stage. The asterisks denote statistically significant differences (Mann-Whitney test); *p < 0.05; **p < 0.01; ***p < 0.001.

At stage I of infection, no bacteria were detected in the blood (Fig. 3D,E). However, 57% of mice (n = 12/21) were positive in the plasma for LF detection and 28% (n = 9/32) for EF (Fig. 3E). When LF and EF positive mice were combined, 62% were positive (n = 13/21) meaning that LF was the most sensitive detection parameter. Plasma LF level was circa 1.7 ng/ml (mean, n = 12), while EF concentration was only 4.6 pg/ml (mean, n = 9) (Fig. 3A,B; Table 2). Interestingly, LF and EF could be detected as early as 30 min in 5 and 2 mice out of nine respectively, showing a rapid process for the dissemination of the toxins far from their site of production.

At stage II (BLI signal detected only in the ear cutaneous tissue), *B. anthracis* was detected in 11% of the mice (n = 3/28) (Fig. 3E) while the proportion of mice positive for EF or LF increased to 50 and 85% respectively. When combined, 92% of the mice were positive for EF and/or LF (Fig. 3E). LF level presented about a 5-fold increase to 9.2 ng/ml (mean, n = 11) while EF level did not vary considerably as compared to stage I, remaining at around 7.9 pg/ml (mean, n = 11) (Fig. 3A,B; Table 2).

At stage III, 71% of the infected mice (n = 22/31) were positive for *B. anthracis* in blood with an increase of circulating bacteria to 700 CFU/ml compared to stage II (Fig. 3D). Mice positive for EF and LF in plasma samples were 92% (n = 24/26) and 100% (n = 14/14) respectively. A 120-fold increase in circulating EF was observed reaching circa 960 pg/ml (mean, n = 24), while LF plasma concentration did not vary considerably compared to stage II (Fig. 3A,B; Table 2). At stage IV of systemic dissemination, all mice displayed bacteremia and were positive for EF (Fig. 3D,E); bacterial load in the blood still increased by 180-fold to 14,600 CFU/ml. EF plasma concentrations remained at a similar level compared to the previous stage, whereas LF level decreased by a 4-fold (Fig. 3A,B; Table 2). At stage V, LF level still increased by a 300-fold, reaching around 1,070 ng/ml (mean, n = 6), whereas EF concentration displayed a 10-fold increase (mean, n = 13), (Fig. 3A,B; Table 2).

These dynamics of LF and EF plasma levels were reflected in the variations of the LF/EF ratio (Fig. 3C; Table 3). At stages I and II, due to a high LF and low EF concentration, the ratio LF/EF was around 1,000 (mean, n = 5) (Fig. 3C; Table 3). At stage III and IV, the ratio LF/EF fell to 81 (mean, n = 12) and 3 (mean, n = 7) respectively, reflecting the high increase of EF concentration and the stable or decreasing LF levels. At stage V, the ratio slightly increased to circa 60 (mean, n = 6), consequence of a higher increase of LF versus EF concentration.

For the first time, we show an early detection of *B. anthracis* active EF and LF in the plasma in the first hours of a cutaneous infection, while there were no circulating bacteria in blood and no BLI signal detectable at the initial site of infection.

### In the absence of PA, rapid diffusion of LF and EF into the blood circulation

We have shown that active LF and EF reach the plasma compartment early during infection with the 9602WT-*lux B. anthracis* strain. This could have potential pathological consequences. To address the involvement of PA, i.e. its ability to bind LF and EF and to interact with the ANTXR in the tissues, in the extent of LF and EF diffusion, we conducted a similar analysis of active LF and EF quantification during a cutaneous infection with a PA-deleted bioluminescent strain (9602P-*lux*)^26^.

**Figure 4:**
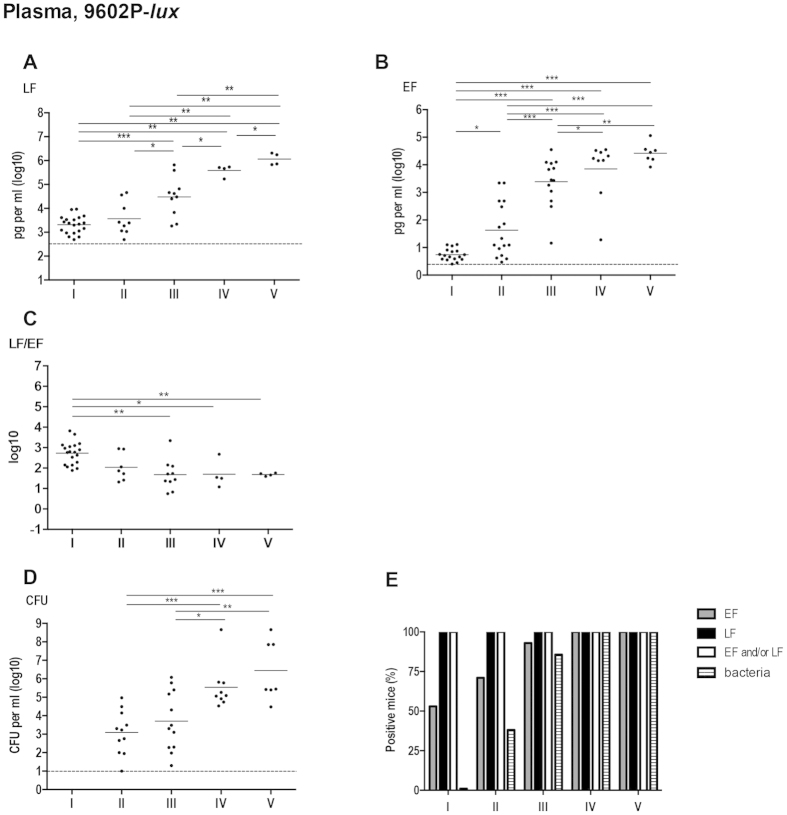
*In vivo* dynamics of LF and EF circulation in plasma during cutaneous infection with the PA-deficient 9602P-*lux* strain. Same experimental conditions as in Fig. 3.

Detection of 9602P-*lux* in the blood and the dynamics of the plasma bacterial load showed a similar pattern as with the 9602WT-*lux* strain at all stages of infection. Bacteria appeared in the bloodstream at stage II (Fig. 4D), in 38% (n = 11/29) of the infected mice (Fig. 4E); the percentage of mice in bacteremia increased to 86% (n = 12/14) at stage III, but with a similar bacterial load. Increase of the bacterial burden started after stage III and reached around 2.8 × 10^6^ CFU/ml at stage V (mean, n = 7). Interestingly, active LF was detected in all mice as soon as stage I (Fig. 4E), while this was observed only at stage V with the wild-type strain (Fig. 3E). Active EF could be detected in all mice only from stage IV onwards (Fig. 4E), similar as for the wild-type strain (Fig. 3E), though a higher proportion of animals were positive during the first three stages.

Active LF concentration at stage I was circa 2.1 ng/ml (mean, n = 20) despite the absence of bacteria in the bloodstream (Fig. 4A,E; Table 2). Then, LF level increased steadily reaching about 1,150 ng/ml at stage V. The decrease observed at stage IV during infection with the wild-type strain (systemic dissemination with BLI signal in the spleen; Fig. 3A) was not detected with the PA-deleted 9602P-*lux* strain (Fig. 4A); at this stage, LF level was 130-fold higher in mice infected with the 9602P-*lux* than in those infected with the wild-type strain. At stage I, circulatory EF concentration was 5.5 pg/ml (mean, n = 17); it then increased to around 40 pg/ml (mean, n = 16) at stage II, and 2,400 pg/ml (mean, n = 13) at stage III, reaching a plateau from stage IV, at around 14,000 pg/ml at stage V (mean, n = 7) (Table 2). Interestingly, there was no difference in EF concentrations in the plasma between the 9602P-*lux* and the 9602WT-*lux* strains during the entire infection process.

Active LF levels were thus still higher than EF levels, but EF concentration increased by a 4,800-fold between stages I and V, compared to a 560-fold increase for LF. The corresponding LF/EF ratio was 530 (mean, n = 19) at stage I, and decreased during infection down to 50 (n = 4) at stage V (Fig. 4C; Table 3).

**Figure 5 f5:**
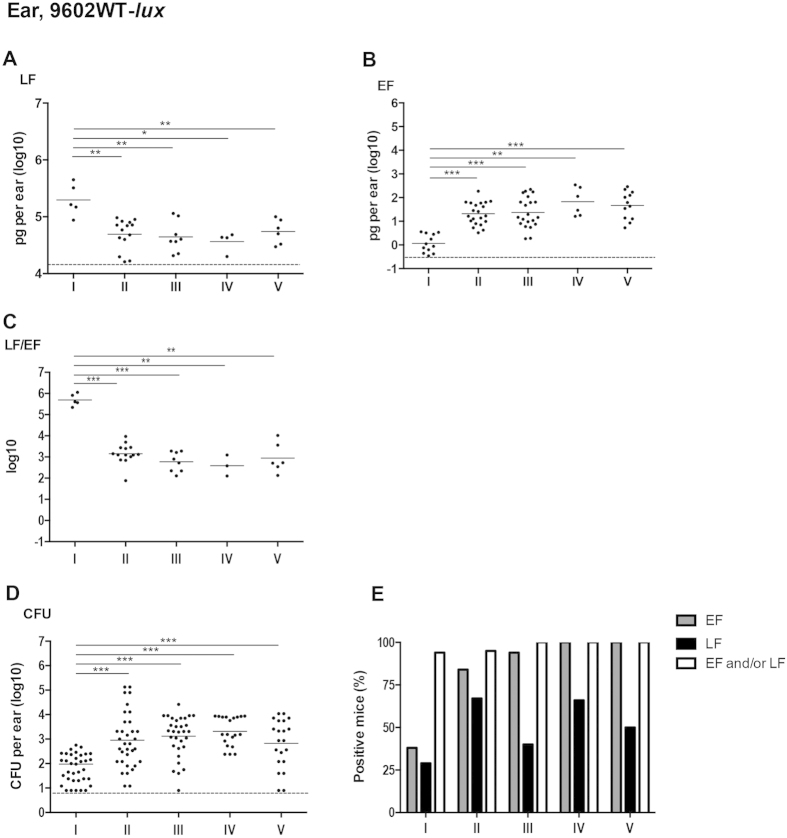
*In vivo* kinetics of production of LF and EF in the inoculated ear cutaneous tissue during infection with the PA-deficient 9602P-*lux* strain. Same experimental conditions as in Fig. 2.

Considering that active LF and EF diffuse from the initial site, at least during the first stages of infection, they were quantified in the ear cutaneous tissue. During the stages I to III, the bacterial load of 9602P-*lux* (Fig. 5D) significantly increased (p < 0.0001) from circa 1 × 10^2^ CFU/ear (mean, n = 26) to 5 × 10^4^ CFU/ear (mean, n = 13) and then, did not vary significantly until stage V. It should be noted that the bacterial burden in the ear cutaneous tissue was higher in absence of PA at stages III (BLI in the dLN), IV (BLI in the spleen) and V (septicemic terminal stage) during infection with the 9602P-*lux* strain than with the 9602WT-*lux* strain (Figs 2D and 5D).

At stage I, 30% of mice were positive for LF (n = 3/10) and 55% for EF (n = 16/29) in the ear cutaneous tissue (Fig. 5E). Thereafter, all mice were positive for LF from stage III and EF from stage II. For comparison, with the wild-type strain, all mice were positive for EF only from stage IV and a maximum of 67% of mice were positive for LF detection (Fig. 2E).

Active LF and EF amounts at stage I were circa 23 ng/ear (n = 3) and 5.3 pg/ear (mean, n = 16) (Fig. 5A[Fig f5]B; Table 2) respectively. Then, LF levels increased at stage II, reaching 166 ng/ear (mean, n = 7). Similarly, a strong increase in the EF levels was observed at stage II and at stage III, reaching a plateau of 400 pg/ear. When compared to the levels detected during infection with 9602WT-*lux*, i.e. in the presence of PA, the levels of active LF at stage II, and the levels of EF from stage I to V, were thus higher in the absence of PA in the cutaneous infected tissue (Figs 2A,B and 5A,B; Table 2). These higher values throughout the infection, especially for active EF, were reflected in the variations of the LF/EF ratio (Fig. 5C; Table 3). At the first stage of infection, the LF/EF ratio was around 14,700 (n = 3). This ratio decreased significantly between stages I and IV (p = 0.0286) with a mean ratio of 31 (n = 4) when the spleen was bioluminescent. At stage V, the mean ratio was 190 (n = 5).

## Discussion

Our study is the first to provide insight into the *in vivo* kinetics of the amounts of both functional LF and EF that are present at the initial site of infection and in the blood during a cutaneous infection with a fully virulent wild-type *B. anthracis* strain in immuno-competent mice. These data are of paramount interest, as very little is actually known on the ratio of EF and LF concentrations *in vivo*, though numerous data are available on the effects of LeTx and EdTx at the cellular, tissue and animal levels (for review^16^,^27^). Previous studies mainly focused on LeTx considered as the major toxin of *B. anthracis*, as it was historically the first factor to be shown to be lethal^28^. But interestingly, EdTx is lethal at lower doses (20–30 μg) than LeTx (40–100 μg) in mice (for review^29^).

In our study, highly sensitive assays detecting the enzymatic activity of each toxin factor were used, enabling an approach of the accurate physiological toxin concentrations in animals throughout a cutaneous infection. We have previously developed a sensitive technique based on the measurement of the enzymatic activity of EF through a highly sensitive immunoassay^25^. We adapted the technique for the measurement of the enzymatic activity of LF coupled to MS setup by Barr’s group^24^; we favored carrying out the enzymatic assays of LF and EF directly in the biological samples, without added purification steps, to enable precise and comparable measurements of the respective amounts of EF and LF. Such approaches have been used to specifically detect and quantify *B. anthracis* EF or LF in plasma from human and various animal species and in serum of macaque^24^,^25^.

To circumvent the naturally asynchronous pattern of the infection and its initial asymptomatic character, we took advantage of the power of BLI in mice to approach the spatial dynamics of bacterial dissemination; we were thus able to define and analyse discrete homogenous stages of the infection. To obtain the most complete vision of the events occurring during infection, we defined five stages for the infection, from the asymptomatic (no BLI signal, no clinical signs) to the terminal stage of anthrax. Using non-encapsulated toxinogenic strains in a model of cutaneous infection in the immuno-compromised A/J mice, Weiner and coworkers also stratified the infection by using BLI. Three stages were defined, the first stage being already associated with detection of a bioluminescent signal in the ear tissue^30^.

In a cutaneous infection, toxin production at the initial site of inoculation of wild-type *B. anthracis* is crucial for the fate of the host^3^,^31^. We show that active LF and/or EF were detected in 94–95% of the infected mice as soon as stage I. This is in agreement with published reports on the rapidity of germination triggering and toxin production. *pagA* mRNA is detected as soon as 15 min after germination triggering^32^. Extensive germination occurs as early as 15 minutes after inoculation in the ear cutaneous tissue 2, or 1–3 h after inoculation^33^, and in the absence of eukaryotic cell contact^2^,^33^.

Interestingly, we detected active LF and EF in the bloodstream as early as stage I while no bacteria could be isolated in the blood circulation. This shows that *B. anthracis* toxin factors can reach the blood vascular system independently of the toxin-secreting bacilli while the bacterial load in the ear cutaneous tissue remained below the detection threshold of the BLI system^26^. At this stage of infection, it was shown that bacterial multiplication occurred only at the initial site of infection, while the small population of spores having reached the dLNs remained as spores^2^. The active LF and EF found in the blood compartment thus originated from production within the infected ear tissue. This may have implications for the pathophysiology of the infection, as toxins may exert their activity at distance at an early time point of infection, and for early diagnosis. Further studies are needed to explore this aspect.

From stage I, the dynamics of circulating LF and EF followed a triphasic profile, i.e. an increase followed by a plateau (and a slight decrease for LF) and a terminal increase at stage V. This pattern was delayed for EF from stage III. Previous studies have focalised only on LF dynamics. During inhalational anthrax in rhesus macaques, a triphasic kinetic profile was also observed for active LF with an increase in concentration between 24 h and 48 h, followed by a transient decrease, and then a dramatic terminal increase again at 120 h^34^. During cutaneous infection with non-encapsulated toxinogenic strains in the immunocompromised A/J mice, circulating active LF in serum was at 0.894 ng/ml when BLI was detectable at the site of inoculation^30^; its concentration increased when the dLN displayed luminescence, and reached 1,973 ng/ml when mice were in septicemia; no data were available at the stage of the bioluminescent spleen. The LF values measured in our study at equivalent stages, but during infection with an encapsulated toxinogenic wild type *B. anthracis* strain, were 9.2 ng/ml and 1,070 ng/ml respectively.

The general trends and magnitude appear consistent between these studies. However, comparison of EF and/or LF amounts reported in our and previous studies^3^,^21^,^30^,^34^,^35^ are quite delicate as the techniques used for quantification as well as the animal models are different; *i.e.* for example detection of total LF and EF immunoreactive proteins by western-blot; infection with non-encapsulated *B. anthracis* that presents a widely different pattern and dynamics of infection^1^,^26^,^36^.

As PA binds LF and EF *in situ* and the heptamer complex interacts with the anthrax toxin receptors on the cell surface in the infected tissue, all this can influence LF and EF levels in the tissues and their ability to diffuse at distance, by consumption effects. To approach how the presence of PA can influence the levels of local and circulating LF and EF, we characterised the diffusion of active LF and EF into the blood during infection with a PA-deleted *B. anthracis* strain.

The absence of PA did not modify the EF levels in the bloodstream compared to the wild type strain. LF dynamic pattern was also unchanged except at stage IV, when the spleen displayed BLI; LF amount still increased in the absence of PA while it fell with the 9602WT-*lux* strain. This indicates that this decrease, also observed in macaques^34^, was dependent on PA. It might be speculated that LF is sequestered in the tissues at this specific stage while binding to PA and on the receptors, thus suggesting a potential time window of acute toxicity of LF acting in target organs. Bacterial burden was also similar in the absence of PA.

This indicates that PA was not implicated in the early diffusion of LF and EF in the bloodstream and in the levels of EF and bacteria along infection.

In the ear cutaneous tissue at stage I, the levels of LF and EF were ten times lower and eight times higher respectively in the absence of secreted PA. It may be hypothesised that PA retains LF in the ear cutaneous tissue and, when PA is absent, more LF is able to diffuse from the initial site of infection. Indeed, either in ear tissue or in plasma, the percentage of mice positive for EF or LF was higher in absence of PA and occurred more rapidly, suggesting an increased availability of the factors that could then be detected.

Our present data are relevant with the few available observations made in human anthrax cases. It was indeed reported that detectable LF levels were found in the blood of 50% of cutaneous anthrax cases at an early stage when the infectious process remained localized at the initial lesion site^35^. In cases of human cutaneous anthrax, PA was detected in the blood in 50 to 100% of the patients after only 2–6 days after the diagnostic^35^,^37^. Furthermore, in another study of human cutaneous anthrax, modifications of brain signal were found by using magnetic resonance imaging, despite the infection remaining localised to the skin^38^; this strongly suggested an early diffusion of toxins able to act at distance. This could be of pathophysiological consequence at the initial stage of anthrax and warrants further studies.

Our data further suggest that EF may exert its toxic activity preferentially at distance, as it seems more able to diffuse. For example, EdTx can sensitize mice to LeTx in LeTx-resistant mouse strains^39^ and increase the sensibility of the host to the anthrax toxins through upregulation of the toxin receptors on macrophages *in vitro*^40^. EdTx enables dissemination through/from the dLN^4^. This means that even produced at a low dose, EdTx may be a significant virulence factor, as both toxins can cooperate^41^. Nevertheless, even the small proportion of active LF that reached the blood compartment could be high enough to exert its toxic activity at distance. Indeed, a proportion of 0.3% to 10% of the bacterial population of a non-encapsulated toxigenic *B. anthracis* strain that produce LF was sufficient for successful colonization and dissemination at the early stages of cutaneous infection in the A/J mouse model^31^. Taking into account our data and the literature, EF and LF thus can diffuse into the lymphatic system from the initial site of infection and transit through the dLNs before reaching the blood vascular system at a very early time point of infection. During this journey, toxins could exert their toxic effects on the immune and lymphatic systems. It is thus proposed that circulating toxins diffusing from the site of infection might be capable of manipulating the innate immune system^17^,^18^,^19^ in sites distal from areas with bacterial growth at an early time point of the infectious process.

All so far published studies mainly quantified LF. Very few data are available on both LF and EF and these covered amounts of total factors. Ratios of 20:5:1 and 10:5:1 were reported for PA:LF:EF after *in vitro* bacterial culture of a toxinogenic non-encapsulated Sterne strain; quantification was either of total proteins^42^ or of beta-galactosidase activity of transcriptional fusions^43^. An LF/EF ratio of 5:1 was observed *in vivo*, the levels of total PA, LF and EF being quantified by western blot in serum of rabbits at the terminal septicemic stage of anthrax^21^. All these studies are informative of a late stage of bacterial multiplication, either *in vitro* or *in vivo*, but none quantified the level of enzymatically active EF and LF *in vivo*.

The present study is the first to approach this crucial aspect of anthrax, i.e. quantifying both active EF and LF during an infection. We provide the first insight into the ratio of active LF and EF produced along a natural infection with a fully virulent strain. Due to the important time-dependent variations of EF and LF production *in vivo*, this ratio displayed wide fluctuations during the entire infectious process. Nevertheless, some emerging points can be stressed. At the initial stages of infection, due to extremely low levels of detectable active EF, the active LF/EF ratio was found to be of 5 orders of magnitude in the initial cutaneous site of infection, and 3 orders in the plasma. Then, this ratio stayed between 400 and 1,500 in the ear tissue, and 50 to 1,500 in the plasma. Even at the terminal septicemic stage of infection the ratio remained around 50. Interestingly, only at stage IV (spleen bioluminescent, no signal in the lungs) could an LF/EF ratio of 3 be observed. We have previously shown that, during infection with wild-type *B. anthracis*, LeTx-induced lesions were first observed in the spleen when few bacteria were present in the infectious foci, while EdTx-induced lesions later appeared when the number of bacteria increased in the foci^4^. This is in agreement with our results that demonstrate that LF is produced in excess compared to EF at the first stages of the infection and with a decrease of the ratio of enzymatic active LF/EF along infection. In the plasma, at stage IV, we can even detect more EF than LF in some mice. For the goal of applying of these assays to early diagnosis of anthrax, detection of both LF and LF should be considered, since, though higher amounts of LF are produced and detected, the higher sensitivity of the EF assay enables detection of lower quantities of EF.

**Figure 6:**
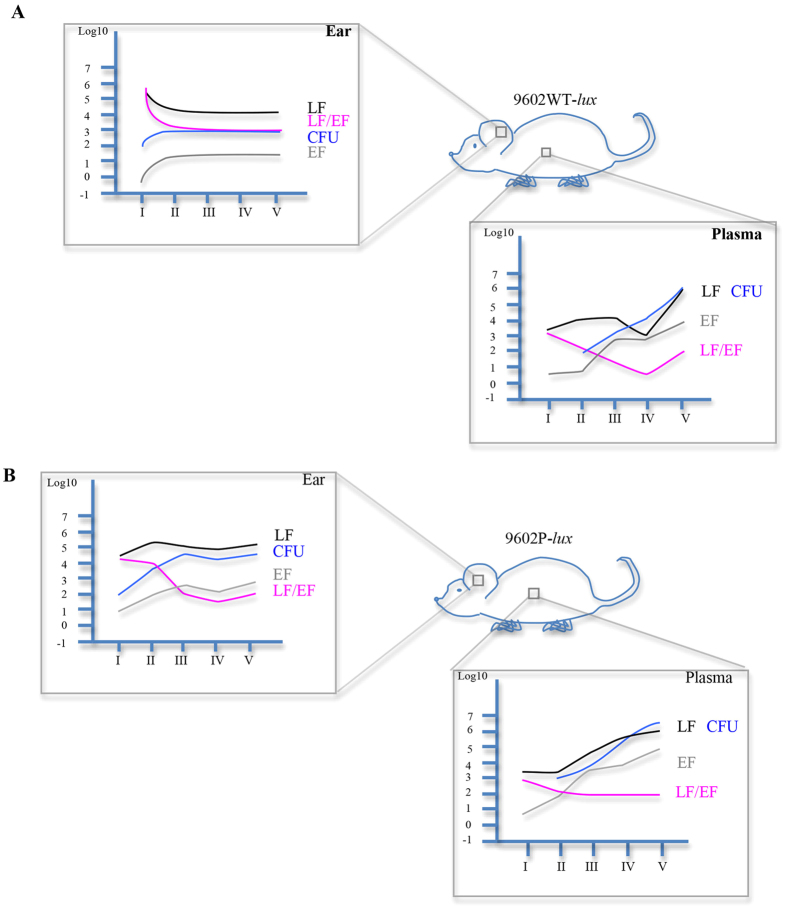
Shematic view of what happens locally and in the bloodstream during cutaneous anthrax infection. The sheme (**A**) and the sheme (**B**) resume data obtained in mice infected with the 9602WT-*lux* strain and the 9602P-*lux* strain respectively, in the inoculated ear and in the plasma for EF, LF concentrations (mean, pg/ear and pg/ml in the ear and in the plasma respectively), the ratio LF/EF (mean) and for the CFU (mean, log10 CFU/ear and CFU/ml in the ear and in the plasma respectively), during time course of infection.

In summary, our data, using highly sensitive assays, provide a novel understanding into the kinetics of LF and EF production along cutaneous anthrax. We show that toxin factors can diffuse at distance at a very early stage of the infectious process with a predominant amount of LF over EF; PA played an important role in the control of the levels of LF, EF and bacterial load in the site of infection contrary to what happen in the bloodstream, except at stage IV for LF (Fig. 6). Furthermore, our data emphasise the highly complex interactions between *B. anthracis* and its various tissular environments during its journey from the initial site of entry to systemic dissemination and colonisation of the infected host. This is in keeping with our previous report showing that, during inhalational infection, the infectious process can develop along two different patterns, either EdTx- or LeTx-dominated, for each individual infected host^4^. Our results thus have important implications for the pathophysiology of the infection. Exploration of the activities of both toxins *in vivo* or *in vitro* should take into account these parameters to come closer to the pathophysiology of the host-pathogen interactions during anthrax. Finally, as the toxins could be detected earlier than expected, this paves the way for early diagnosis of anthrax before the pathophysiological consequences of toxin actions on tissues and organs emerge.

## Materials and Methods

### Chemicals and Reagents

Recombinant anthrax EF from *B. anthracis* was from Quadratech Diagnostics (Epsom Surrey, U.K.). Adenosine 5′-triphosphate (ATP) disodium salt hydrate, adenosine 3′, 5′ cyclic monophosphate, sodium periodate, rhamnose, were from Sigma-Aldrich (St. Louis, MO). Calmodulin human recombinant (rCaM) was from Enzo Life Sciences (Villeurbanne, France). cAMP antiserum (polyclonal antibodies), cAMP acetylcholinesterase (AChE) enzymatic tracer, Ellman’s reagent, cAMP standard, and acetic anhydride used in the EIA studies were from Spi-Bio (Montigny-Le-Bretonneux, France). Recombinant anthrax lethal factor (LF) from *B. anthracis* was from List Biological Laboratories (Campbell, California). Peptide substrate (H-Ser-Lys-Ala-Arg-Arg-Lys-Lys-Val-Tyr-Pro-Tyr-Pro-Met-Glu-Asn-Phe-Pro-Pro-Ser-Thr-Ala-Arg-Pro-Thr-OH) cleaved by LF, N-terminal/C-terminal substrate (H-Ser-Lys-Ala-Arg-Arg-Lys-Lys-Val-Tyr-Pro and Tyr-Pro-Met-Glu-Asn-Phe-Pro-Pro-Ser-Thr-Ala-Arg-Pro-Thr-OH) and internal standards were from Bachem (Bâle, Switzerland). The sequences of these peptides are the same as the sequences of peptides used by Boyer *et al.*^24^

### Ethics statement

All the animal experiments described in the present study were conducted at the Institut Pasteur according to the European Directive 2010/63/UE and were approved by the Institut Pasteur animal care and use committee. All efforts were made to minimize suffering. The animals were housed in animal facilities of the Institut Pasteur licensed by the French Ministry of Agriculture and complying with the European regulations. The protocols were approved by the Institut Pasteur Safety Committee, and Animal Experimentation Ethics Committee (CETEA 2013-0088/MESR 01168.01).

### Animal and bacterial strains

Female BALB/c mice (6 to 10 weeks old) were purchased from Charles River (L’Arbresle, France). The luminescent *B. anthracis* strains used were the wild-type clinical isolate 9602WT-lux (EF+LF+PA+), the isogenic derivative mutant inactivated in the *pagA* gene 9602P-lux (EF + LF + PA-), constructed by insertion of the *luxABCDE* operon of *Photorhabdus luminescens* under the control of the *pagA* promoter as described previously^4^,^26^. The isogenic derivative mutant inactivated in the *cya* and *lef* genes 9602LC (EF-LF-PA+) was used as negative control of production of EF and LF. 9602LC strain was constructed with toxin-inactivation mutants in the *cya* gene [9602C (LF+PA+)] and in the *lef* gene [9602L (EF+PA+)] by insertion of a spectinomycin and erythromycin resistance cassette, respectively, by conjugation as described previously^4^.

### Mouse infection

Anesthetized mice were challenged with spores of each bacterial strain into the ear pinna as described previously^26^. The inoculum size was verified retrospectively by plating 10-fold serial dilutions on Brain Heart Infusion (BHI) (Difco, Detroit Michigan) agar plates (2.73 ± 0.32 log10 CFU - mean ± SD, n = 24; 2.46 ± 0.35 log10 CFU - mean ± SD, n = 19; 2.53 ± 0.33 log10 CFU - mean ± SD, n = 8 for the 9602WT-lux, 9602P-lux and 9602LC strains respectively).

Inoculated ear cutaneous tissue was homogenized in 0.4 ml of ice-cold water + 10% proteases inhibitors mix (Roche). Blood was collected with calciparine 50 IU per ml and proteases inhibitors mix. All samples were centrifuged at 7000 rpm for 4 min. Plasma and ear samples were then filtered on Ultrafree-MC centrifugal Filter Units 0.22 μm (Dominique Dutscher) and conserved at −20 °C for ulterior LF and EF activity assay.

Before filtration, a volume of each sample was serially diluted and plated on BHI agar plates for bacterial enumeration.

### Bioluminescent imaging

Bioluminescent imaging was acquired using an IVIS 200 system (Xenogen Corp, Hopkinton, MA, USA) according to instructions from the manufacturer. Analysis and acquisition were performed using Living Image 3.0 software (Xenogen Corp) as previously described ]^4^.

### LF enzymatic activity assay through LC-MS/MS quantification

The enzymatic activity of LF was assayed in 100 μl reaction buffer (RB), containing 40 mM Hepes buffer pH 7.3, 1 mM DTT, 20 μM CaCl_2_, 10 mM MgCl_2_, 20 μM ZnCl_2_, 1 mg/ml BSA, proteases inhibitors mix (Roche), and 1.24 nM substrate peptide LF. Standards were prepared by serial dilution of rLF from 10 ng per ml to 0.05 ng per ml in matrix from non-infected animals (plasma, cutaneous ear sample) in the presence of proteases inhibitors mix with the same matrix dilution as in the tested sample. Blank control corresponded to no rLF. Adequate dilutions of the samples from infected animals in RB were used to ascertain to be within the standard curve. Standards and samples were incubated with RB buffer and substrate peptide at 37 °C for 10 min.

After elimination of the proteins through precipitation with acetonitrile (ACN) containing 1% formic acid, the LF reaction samples were dried by vacuum centrifugation to eliminate solvents. The N-Ter and C-Ter peptides produced by cleavage of the substrate peptide by LF were then suspended in mobile phase A containing 10% acetonitrile, 1% trifluoroacetic acid, and 0.1% formic acid. After centrifugation, the supernatant (20 μl) was injected into a liquid chromatography system coupled to a triple quadrupole mass spectrometer (LC-MS/MS). Chromatography was achieved on a Zorbax SB-C18 column (150 × 2.1 mm i.d., 5 Nm particle size, 300 Å porosity) from Agilent Technology (Palo Alto, CA, USA) with a gradient elution of mobile phase A (water with 0.1% formic acid) and mobile phase B (acetonitrile with 0.1% formic acid). The mass spectrometer was a TSQ Quantum Ultra (Thermo Scientific, San Jose, USA) operated in the positive ion mode and coupled to the HPLC column via an electrospray interface. Results were analyzed with Thermo Scientific™ Xcalibur software. To keep as close as possible to the pathophysiological events occuring during an infection, background signal and threshold were determined from plasma and cutaneous ear tissue samples of animals infected with an encapsulated *B. anthracis* strain devoid of LF and EF, i.e. inactivated in the *lef* and *cya* genes.

### EF enzymatic activity assay

The adenylyl cyclase EF activity was measured by assessing the production of cAMP using a competitive enzyme immunoassay (EIA) as described previously 25. Briefly, the assay consists of three steps. The first one is the enzymatic reaction assay, i.e. each sample from infected animals (plasma, cutaneous ear tissue) was diluted in 20 mM Hepes buffer pH 7.2 with 0.5 mg/ml bovine serum albumin, 10 mM MgCl2, 10 μM CaCl2, 1 mM ATP and 10 μM CaM; adequate dilutions were used to ascertain to be within the standard curve. Standards were prepared by serial dilution of the EF from 100 to 1.5 pg/ml in matrix of non-infected mice (plasma, cutaneous ear sample) with the same matrix dilution as in the tested sample. Blank control corresponded to no rEF. The adenylyl cyclase activity was then assayed for 30 min at 30 °C. The second step was elimination of ATP interference in the assay and increase of cAMP detection sensitivity by chemical transformations (production of acetylated cAMP)^25^. The third step was the EIA: acetylated cAMP was in competition with cAMP enzymatic tracer, cAMP coupled to acetylcholinesterase (AChE), for binding to anti-cAMP polyclonal antibodies, for 1 h 30 at room temperature. The amount of tracer bound to the anti-cAMP antibody site was then measured by incubation with AChE substrate (Ellman’s reagent) at room temperature overnight. The absorbance was recorded at 405 nm using a spectrophotometer microplate reader Multiskan Ascent (Labsystem). The response is inversely proportional to the concentration of cAMP in the sample. To keep as close as possible to the pathophysiological events occurring during an infection, background signal and threshold were determined from plasma and cutaneous ear tissue samples of animals infected with an encapsulated *B. anthracis* strain devoid of LF and EF, i.e. inactivated in the *lef* and *cya* genes. No intrinsic adenylyl cyclase activity was detected in the infected samples in the absence of calmodulin, and the endogenous ATP and cAMP concentrations in the infected samples did not interfere with the EF assay.

cAMP degradation was verified by conducting EF detection with and without rolipram, a phosphodiesterase inhibitor, in ear and plasma samples. Same EF concentrations were obtained showing that there was not significant cAMP degradation due to manipulation.

### Statistical analysis

Statistical analysis and graphing were performed with GraphPad Prism software (version 4.0; GraphPad Software, San Diego, CA). Statistical significance was determined by Mann-Whitney test. *P* < 0.05 was set as the statistically significant threshold.

## Additional Information

**How to cite this article**: Rougeaux, C. *et al.* *In vivo* dynamics of active edema and lethal factors during anthrax. *Sci. Rep.* **6**, 23346; doi: 10.1038/srep23346 (2016).

## Figures and Tables

**Table 1:** Basal values of LF and EF assays in plasma and ear matrix during cutaneous infection with the LF- and EF-deleted *B. anthracis* 9602LC strain.

Time after infection	Plasma			Ear		
	CFU^a^	EF assay^b^	LF assay^b^	CFU^c^	EF assay^d^	LF assay^d^
1 h	<1 (22)	0.14 ± 0.05 (16)	1.91 ± 0.11 (9)	2.18 ± 0.73 (17)	−2.92 ± 0.06 (18)	3.96 ± 0.07 (7)
41 h	6.03 ± 2.26 (12)	−0.1 ± 0.11 (15)	1.89 ± 0.10 (12)	4.65 ± 0.7 (20)	−2.79 ± 0.22 (24)	3.64 ± 0.13 (17)

Data are expressed as mean ± SEM, ^a^log_10_ CFU/ml, ^b^basal values in the EF and LF assays log_10_ pg/ml, ^c^log_10_ CFU/ear tissue sample, ^d^basal values in the EF and LF assays log_10_ pg/ear tissue sample; number of samples (n) in brackets. Mice were inoculated into the subcutis of the ear pinna with spores of the LF- and EF-deleted 9602LC strain. At 1 h and 41 h of infection, total CFU were enumerated in the plasma and the inoculated ear cutaneous tissue (threshold values 1 log_10_ in the blood and 0.9 log_10_ in the ear tissue). Basal values in the EF and LF assays were determined in the same samples as described in the Material and Methods section. From these values obtained in the matrix from mice infected with an LF- and EF-deleted *B. anthracis* strain, thresholds for EF and LF were thus determined to be 0.4 and 2.6 log_10_ pg/ml respectively in the plasma, and −0.47 and 4.2 log_10_ pg/ear respectively in the ear cutaneous tissue.

**Table 2:** *In vivo* dynamics of LF and EF production in plasma and ear cutaneous tissue during infection with *B. anthracis*.

Infection with	Sample		Stage of infection stratified along the bioluminescence signal				
			I	II	III	IV	V
9602WT-*lux*	Ear cutaneous tissue	LF^a^	5.3 ± 0.13 (5)	4.69 ± 0.07 (14)	4.64 ± 0.1 (8)	4.56 ± 0.09 (4)	4.74 ± 0.09 (6)
		EF^a^	0.06 ± 0.11 (12)	1.32 ± 0.1 (21)	1.37 ± 0.14 (21)	1.82 ± 0.24 (6)	1.67 ± 0.17 (12)
		CFU^b^	1.98 ± 0.2 (36)	2.95 ± 0.19 (34)	3.12 ± 0.15 (31)	3.32 ± 0.13 (20)	2.83 ± 0.22 (21)
	Plasma	LF^c^	3.23 ± 0.12 (12)	3.96 ± 0.16 (11)	4.14 ± 0.17 (14)	3.52 ± 0.16 (8)	6.03 ± 0.48 (6)
		EF^c^	0.66 ± 0.08 (9)	0.92 ± 0.08 (11)	2.98 ± 0.18 (24)	3.29 ± 0.27 (9)	4.28 ± 0.13 (13)
		CFU^d^	<1	1.91 ± 0.33 (3)	2.85 ± 0.23 (22)	4.16 ± 0.4 (12)	6.32 ± 0.28 (11)
9602P (Δ*pagA)*-*lux*	Ear cutaneous tissue	LF	4.35 ± 0.08 (3)	5.22 ± 0.11 (7)	4.92 ± 0.15 (12)	4.68 ± 0.14 (5)	5.2 ± 0.26 (6)
		EF	0.72 ± 0.15 (16)	1.79 ± 0.17 (10)	2.61 ± 0.26 (16)	2.34 ± 0.4 (9)	2.97 ± 0.41 (8)
		CFU	2.02 ± 0.15 (26)	3.63 ± 0.37 (9)	4.7 ± 0.15 (13)	4.03 ± 0.48 (9)	4.97 ± 0.43 (9)
	Plasma	LF	3.31 ± 0.08 (20)	3.56 ± 0.23 (9)	4.48 ± 0.27 (10)	5.58 ± 0.12 (4)	6.06 ± 0.13 (4)
		EF	0.74 ± 0.05 (17)	1.63 ± 0.25 (16)	3.38 ± 0.25 (13)	3.85 ± 0.36 (9)	4.42 ± 0.13(7)
		CFU	<1	3.09 ± 0.36 (11)	3.71 ± 0.47 (12)	5.54 ± 0.41 (9)	6.44 ± 0.61 (7)

Data are expressed as mean ± SEM, ^a^EF and LF concentration log_10_ pg/ml of homogenized ear tissue sample, ^b^log_10_ CFU/ml of homogenized ear tissue sample, ^c^EF and LF concentrations log_10_ pg/ml, ^d^log_10_ CFU/ml; number of samples (n) in brackets. Mice were inoculated into the subcutis of the ear pinna with spores of the wild type 9602-*lux* or the PA-deleted 9602P-*lux* strain. At each stage of infection as defined in the results section, EF and LF concentrations, and total bacterial CFU were enumerated in the each sample in the plasma and the inoculated ear cutaneous tissue as described in the Material and Methods section. Threshold values as determined in Table 1.

**Table 3:** *In vivo* dynamics of LF/EF ratio in plasma and ear cutaneous tissue during infection with *B. anthracis*.

Infection with	Sample	LF/EF ratio at each stage of infection stratified along the bioluminescence signal				
		I	II	III	IV	V
9602WT-*lux*	Ear cutaneous tissue	5.51 ± 0.22 (5)	3.15 ± 0.13 (14)	2.77 ± 0.17 (8)	2.61 ± 0.2 (4)	2.95 ± 0.29 (6)
	Plasma	3.17 ± 0.31 (5)	2.69 ± 0.28 (5)	1.91 ± 0.28 (12)	0.49 ± 0.37 (7)	1.7 ± 0.45 (6)
9602P (Δ*pagA)*-*lux*	Ear cutaneous tissue	4.17 ± 0.38 (3)	3.49 ± 0.13 (7)	2.17 ± 0.4 (12)	1.49 ± 0.74 (4)	2.28 ± 0.94 (5)
	Plasma	2.73 ± 0.13 (19)	2.04 ± 0.25 (7)	1.68 ± 0.24 (10)	1.7 ± 0.34 (4)	1.68 ± 0.04 (4)

The LF/EF ratio was calculated for each individual mouse displaying values over the thresholds for both LF and EF in the ear cutaneous tissue and the plasma. Data are expressed as mean log_10_ ± SEM, number of samples (n) in brackets. Same experimental conditions as in Table 2.
