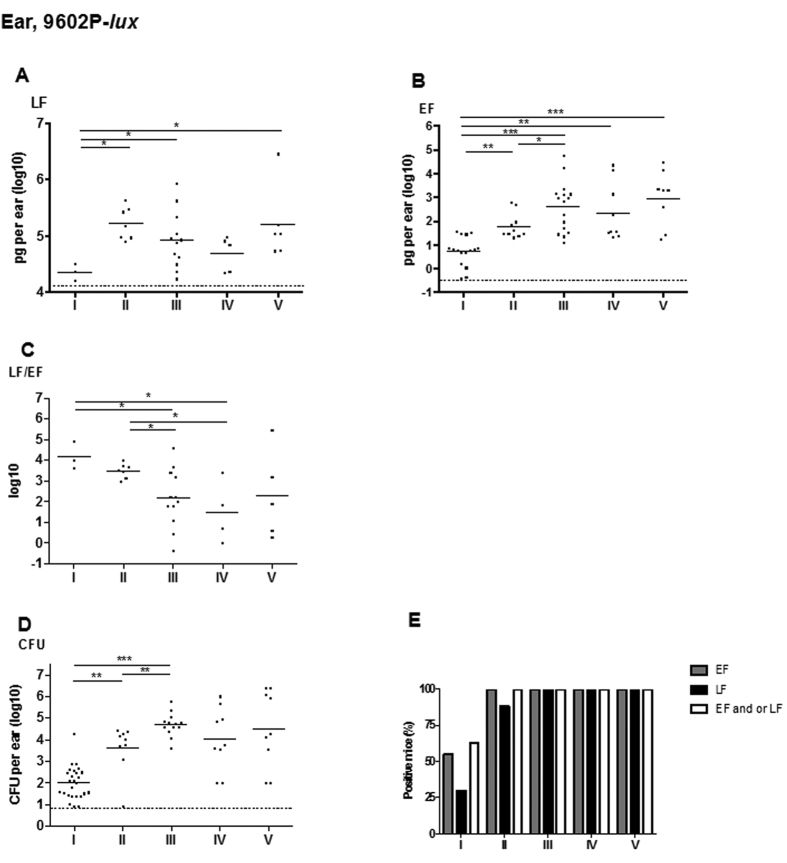# Corrigendum: *In vivo* dynamics of active edema and lethal factors during anthrax

**DOI:** 10.1038/srep39579

**Published:** 2017-02-13

**Authors:** Clémence Rougeaux, François Becher, Eric Ezan, Jean-Nicolas Tournier, Pierre L. Goossens

Scientific Reports 6: Article number: 2334610.1038/srep23346; published online: 03 21 2016; updated: 02 13 2017

In this Article, Figure 5 is a duplication of Figure 2. The correct Figure 5 appears below as Figure 1.

## Figures and Tables

**Figure 1:**